# Subscapular abscesses: A literature review and evidence-based treatment guidelines

**DOI:** 10.1177/17585732231165194

**Published:** 2023-03-20

**Authors:** Isobel V McFarlane, Marcus Wong, Angela Chang Alder-Price

**Affiliations:** 1 Adelaide Medical School, 1066 University of Adelaide, Adelaide, South Australia, Australia; 2 Department of Orthopaedics and Trauma, 3187 Lyell McEwin Hospital, Elizabeth Vale, South Australia, Australia

**Keywords:** subscapular, subscapularis, abscess, pyomyositis

## Abstract

**Background:**

An intramuscular abscess of the subscapularis is a rare phenomenon but important pathology for surgeons to be aware of because clinical deterioration can be rapid and diagnosis difficult. The presentation often mimics other common shoulder pathologies with subacute shoulder pain and stiffness. Early diagnosis, antibiotics and surgical drainage are critical to reduce the spread and joint destruction.

**Methods:**

A search of PubMed and Google Scholar databases identified cases of subscapular intramuscular abscess. Data collected about each case included patient demographics, presentation, pathology, surgical treatment and outcome. The authors report one additional subscapular abscess case.

**Results:**

Data from 17 cases of subscapular abscess were found, 16 in the literature and one case described by the authors. Sixteen of 17 cases (94.1%) presented with shoulder pain and reduced range of motion worsening over a mean of 6.7 days prior to presentation. Surgical approaches utilised included a posterior inferomedial approach, deltoid-pectoral approach and one posterior inferolateral approach.

**Discussion and conclusions:**

From the limited data available regarding subscapular intramuscular abscess, the authors make the following recommendations: (1) Empirical antibiotics covering *Staphylococcus aureus* +/− methicillin-resistant *Staphylococcus aureus*, (2) drainage is indicated in all cases; and (3) tendon-sparing approaches can access an abscess in most locations within the subscapular space.

## Introduction

An intramuscular abscess of the subscapularis is a rare phenomenon with only 16 cases reported in the literature. The presentation of a subscapular abscess, with subacute shoulder pain and stiffness, mimics that of many common shoulder pathologies. Patients often present late, diagnosis can be difficult and clinical deterioration rapid.^1,2^ High clinical suspicion and soft tissue imaging are vital to early diagnosis.^3^ Antimicrobial therapy and prompt surgical drainage reduce the chance of local spread to the glenohumeral joint with resultant joint destruction and haematological dissemination. Given the difficult surgical location of the subscapularis muscle, surgical approaches in the literature have varied greatly, often involving large incisions, tenotomies or extensive dissection of local anatomy.

Currently, no guidelines exist to help guide diagnosis and management of this rare clinical entity. The aim of this study was to conduct a review of the literature surrounding subscapular intramuscular abscess to present the first set of evidence-based treatment recommendations. The authors present an additional case utilising a muscle and tendon-sparing approach to drain the subscapular abscess.

## Methods

A search of the PubMed and Google Scholar databases was conducted to identify cases of subscapular intramuscular abscess. The references of included articles were manually searched to identify additional cases. Cases that contained details of the subscapularis abscess presentation and management were included in this study. Collected data included demographic information, medical comorbidities, presenting signs and symptoms as well as abscess size and identified organism, surgical approach and outcome of treatment. Only articles written in English were included. In addition to the published data, the authors report additional subscapularis abscess cases to add to the data analysis. The data was tabulated to identify any patterns in presentation, treatment and outcome in order to develop treatment guidelines.

## Results

### Review of literature

A PubMed and Google Scholar literature search with the search terms ‘abscess’, ‘pyomyositis’, ‘subscapular’ and ‘subscapularis’, identified 16 relevant articles with 16 cases reported. Details of the 17 cases including the authors’ case are summarised in Table 1.^1–16^

**Table 1.** Summary of cases of subscapular abscess.

Author (year)	Age	Sex	Possible risk factors	Duration of symptoms	Imaging modality	Glenohumeral joint involvement?	Surgical drainage?	Microbiology	Surgical approach
San Joaquin (1980)	15mo	F	-	5 days	-	-	-	Haemophilus influenzae type B	-
Handorf (1983)	19	M	Blunt trauma	6 days	-	Y	N	*Staphylococcus aureus*	*Deceased*
Nowinski (2004)	53	M	-	14 days	MRI	N	Y	*Staphylococcus aureus*	Posterior inferomedial
Saxena (2007)	42	M	T2DM	incidental finding	MRI	N	Y	*Staphylococcus aureus*	Posterior inferomedial
Babayiğit (2009)	7	M	Blunt trauma	21 days	MRI	N	Y	Nil growth	-
Yilmaz (2012) *Case 1 only	9	F	T1DM	7 days	MRI	Y	Y	*Staphylococcus aureus* (MRSA)	Deltoid-pectoral
Giugale (2015)	9	F	-	7 days	MRI	N	Y	*Staphylococcus aureus* (MRSA)	Posterior inferomedial
Koratala (2016)	51	M	T2DM, ESRD with ipsilateral fistula	2 days	CT	N	N	*Staphylococcus aureus* (MRSA)	*Percutaneous catheter drainage*
Christman-Skieller (2017)	23	F	recent IVDU and Hep C	4 days	CT	N	Y	*Staphylococcus aureus* (MRSA)	Posterior inferolateral
Mourkus (2018)	7	M	-	1 day	US/MRI/CT	N	Y	*Staphylococcus aureus*	Posterior inferomedial
Jagernauth (2018)	38	F	Subcutaneous abscess > 1 year ago	4 days	MRI	N	Y	*Staphylococcus aureus*	deltoid-pectoral with conjoint tenotomy
Patel (2018)	38	F	Blunt trauma	4 days	MRI	N	Y	*Staphylococcus aureus*	Deltoid-pectoral with conjoint tenotomy
Furuhata (2019)	67	F	-	2 days	CT/MRI	Y	Y	*Streptococcus pneumoniae*	Deltoid-pectoral with subscapular tenotomy
Fernández Pérez (2020)	44	M	-	10 days	CT	Y	Y	*Staphylococcus aureus* (MRSA)	Deltoid-pectoral with conjoint tenotomy
Park (2020)	32	F	-	6 months	MRI	N	Y	Nil growth	Radical excision of subscapularis
East (2020) * Case 1 only	47	F	T2DM	6 days	CT	N	Y	*Staphylococcus aureus*	Deltoid pectoral with partial dissection of deltoid from humerus
*Authors’ case*	20	F	Recent submandibular cyst	7 days	MRI	Y	Y	*Staphylococcus aureus* (MRSA)	Deltoid-pectoral with tendon sparing

T1DM: type 1 diabetes mellitus; T2DM: type 2 diabetes mellitus; ESRD: end stage renal disease; IVDU: intravenous drug use; CT: computed tomography; MRI: magnetic resonance imaging; US: ultrasound; MRSA: methicillin-resistant *Staphylococcus aureus*.

There were nine reports of abscess found in female patients (52%) and eight found in males (47%). The mean age was 29.8 years (range 15 months to 67 years), with five of the patients (29.4%) under 10 years old. Eight of the patients had factors that predisposed them to the development of an abscess or infection. These included a haematoma from blunt trauma (*n* = 3), poorly controlled diabetes mellitus (*n* = 4), and recent intravenous drug use with a concomitant hepatitis C infection (*n* = 1). All but one patient (94%) was febrile on presentation, and all but one patient presented with shoulder pain and reduced range of motion worsening over a mean of 6.7 days (range 1–21 days) prior to presentation. Four patients (23.5%) presented with an acute pneumonia or septicaemia of unknown origin, with the subscapular abscess, later identified. One patient died approximately 24 h after admission due to cardiac arrest.^2^

Ninety-four percent of patients had at least one elevated blood marker for infection. White cell count and C-reactive protein were both elevated in 82% of the cases. Imaging was used to diagnose and localise the abscess in every case. Eight reports, including the authors’, utilised magnetic resonance imaging (MRI) to confirm the diagnosis, while six used computed tomography (CT) scanning.

S*taphylococcus aureus* was the most commonly identified organism, cultured in 13 of 17 (76.5%) operative samples, with six of these being methicillin-resistant *Staphylococcus aureus* (MRSA). *Haemophilus influenzae* type B and *Streptococcus pneumoniae* were each identified in one patient, and two patients had no growth from the operative samples. All patients with a medical co-morbidity risk factor for infection grew staphylococcus. The diameter of each abscess ranged from 4 to 12 cm (mean 7.6 cm). Nine of the cases (53%) reported an extension of the infection beyond the subscapular fossa. Four involved the glenohumeral joint space, three extended inferiorly, one extended into the axilla, and one extended inferiorly as well as into the axilla and the glenohumeral joint space.

Twelve of the studies reported empirical antibiotic use with six (50%) initially covering Gram-positive organisms and MRSA, and the remaining 50% additionally covering Gram-negative and anaerobic organisms.

Surgical drainage was used in all but two (15 of 17, 88.2%) cases. Of the two cases that did not involve surgery, one patient died before the abscess was identified, and the other was unfit for surgery and a percutaneous CT-guided pigtail catheter was utilised to drain the collection.

Several surgical techniques were used to drain the purulent collections. The inferomedial approach was used in 4 cases (30.7%), the deltopectoral approach with a conjoint tendon tenotomy in 3 cases (23.1%), the deltopectoral approach with other tendon dissections in 3 cases (23.1%), deltopectoral approach with a tendon-sparing approach in 1 case (authors’ case), posterior inferomedial approach in 1 case and a subscapularis radical excision in 1 case (initially suspected to be a sarcoma).

All patients that underwent surgical drainage were discharged with dramatically improved clinical function. 100% of the cases that underwent surgical drainage and from which the outcome was reported, stated an improvement of symptoms and/or inflammatory markers by discharge and almost complete resolution of symptoms at outpatient follow-up. 27% of patients (3 of 11 who reported functional outcomes) had residual stiffness on external rotation at the last follow-up, and physiotherapy was utilised to manage this. The remaining 83% of cases who reported functional outcomes, reported complete resolution of symptoms and function at final follow-up. Three cases underwent multiple operative washouts. Christman-Skieller et al. performed three washouts, the first return to the theatre was protocolled by their institution, and the second was indicated due to high analgesic requirements but no recurrence of infection was found on this third washout.^9^ Mourkus et al. completed a second operation to close the wound after the wound edges were only approximated with sutures initially.^3^ Giugale et al. reported a persistent fever in their patient four days post-operatively; however, intraoperatively only a coagulated haematoma was found, no purulent fluid.^7^ All cases of return to theatre utilised the previous incisions to gain access to the subscapular space.

### Authors’ case

A fit and healthy 20-year-old male presented to his General Practitioner with a simple submandibular cyst that spontaneously drained and was irrigated and packed with gauze. Three weeks later, he presented to the emergency department of a regional hospital with a three-day history of recurrence of the cyst, new left shoulder pain and subjective fevers. He was commenced on intravenous flucloxacillin.

A CT scan of his jaw showed only a superficial infiltrate with no mass or collection, and a CT scan of his left shoulder demonstrated no fluid in the joint. He received an intra-articular steroid injection into the left glenohumeral joint which provided no relief. On day 3 of the admission, the purulent fluid from the cheek cyst grew MRSA, and the antibiotic regimen was changed to intravenous clindamycin and vancomycin. The next day, after three days in this regional hospital, with increasing inflammatory markers (CRP 442 mg/dL, WCC 17.46) and a positive blood culture, he was transferred to a tertiary metropolitan hospital for further investigation.

Upon arrival at the tertiary hospital, the patient was unable to actively move the left shoulder. He had no passive internal or external rotation limited by significant pain but retained some passive forward flexion and abduction of 45°. The shoulder was not warm to palpate nor swollen, but he did have notable tenderness over the anterior and posterior chest wall. He was otherwise comfortable and afebrile with all vital signs within normal limits. He denied intravenous drug use, recent trauma to the left shoulder or chest wall, and had no other symptoms or signs indicating other possible sites of infection.

**Figure 1.**
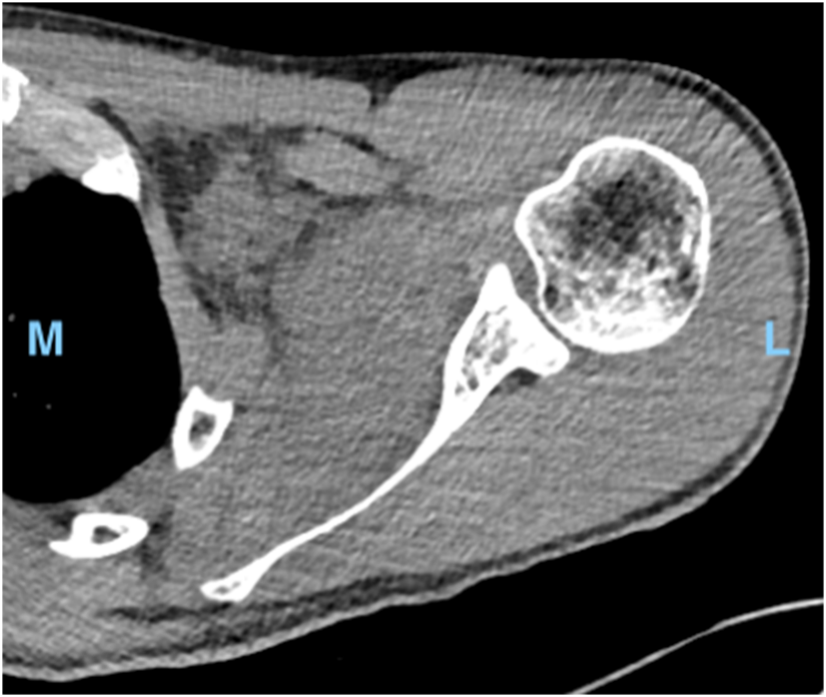
Transverse computed tomography (CT) scan of left shoulder. L, lateral. M, medial.

**Figure 2.**
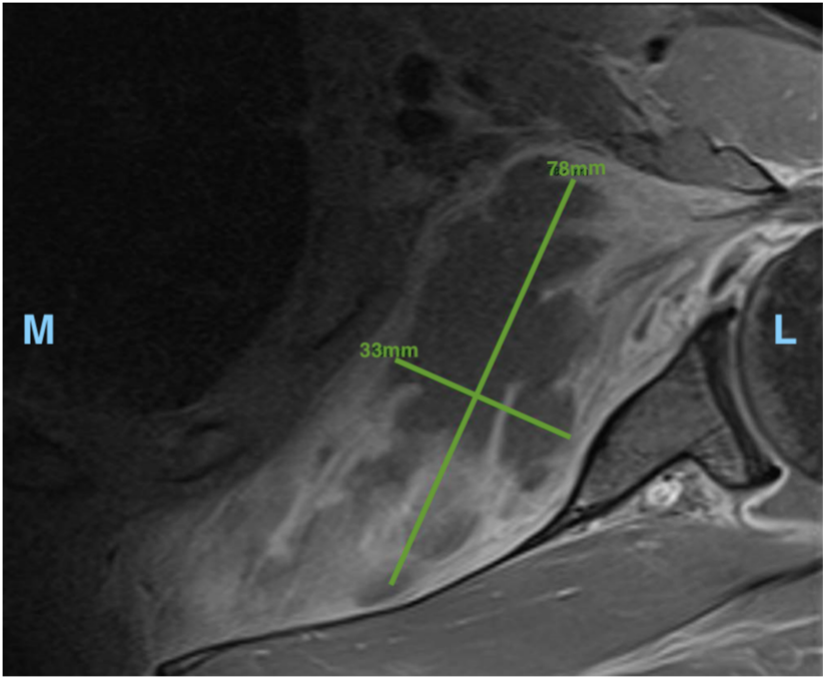
Transverse magnetic resonance imaging (MRI) of left shoulder. Measurements of largest locule (78 × 33 mm). L, lateral. M, medial.

The initial CT was reviewed and irregular soft tissue stranding was noted within the subscapularis muscle that had not previously been reported by the reporting radiologists (Figure 1). A subsequent MRI (Figure 2) demonstrated a large shoulder joint effusion and a multi-loculated fluid collection within the body of the subscapularis, likely representing an abscess. The patient was commenced on intravenous cefazolin and vancomycin and booked for surgical drainage the following day.

A shoulder arthroscopy was first performed using a single standard posteromedial portal to gain access to the glenohumeral joint. A 10 ml of cloudy fluid was drained, and the joint was washed out with several litres of normal saline. The posterior aspect of subscapularis as viewed from inside the joint appeared intact. The procedure was converted to an open deltopectoral approach. The conjoint tendon was found to be adhered to the surrounding tissue and was carefully freed and retracted medially to reveal the underlying subscapularis and its insertion. The subscapularis was split horizontally in its mid portion in line with the muscle fibres, with the split bluntly extended medially with finger dissection. When the subscapular fossa was reached, a large collection of approximately 100 ml of frank pus under pressure was drained. The space was irrigated with saline, and a deep drain was left *in situ*.

MRSA was cultured from the surgical specimens and the patient undertook a course of intravenous vancomycin for two weeks, followed by oral clindamycin for a further six weeks. The wound drain was removed 48 h post-operatively. The patient was encouraged to mobilise the shoulder as tolerated, and by day 3 post-operatively, he was able to actively externally rotate his left arm to 90°. Seven days after drainage of the abscess his serum C-reactive protein had reduced to 28.6 mg/dL, and he was discharged.

At the 2-week follow-up appointment, he had regained a full active range of movement and pain only on resisted external rotation. There were no signs of recurrence of infection and he had returned to work. Six months after his surgical drainage, the patient reported no ongoing issues.

## Discussion

The subscapularis muscle is deep and surrounded by bony structures, consequently, patients with an intramuscular abscess often present late when the collection is large and well established. The shoulder pain and stiffness that commonly present are often mistaken for more common pathologies including septic arthritis or adhesive capsulitis further delaying diagnosis and treatment. A number of the historical cases demonstrate the detrimental result of delayed diagnosis with disseminated infection at presentation, including pulmonary infection and septicaemia, and in one case, subscapular abscess diagnosis was made post-mortem after the patient died of bilateral panlobar pneumonia and septicaemia, secondary to direct extension into the subscapular space, axilla, and musculature of the clavicles and chest wall.^2^ It is important for surgeons to be aware of this pathology to prevent the sequelae of delayed diagnosis and have a low threshold for further imaging with CT or MRI in patients who do not fit the typical pattern of a septic glenohumeral joint or adhesive capsulitis.

This series demonstrates that a subscapularis abscess should be suspected in patients presenting with persistent shoulder pain and elevated blood markers for infection, with a restricted shoulder range of rotation more than flexion or abduction. The symptoms may be of subacute onset (mean time of symptoms onset to diagnosis being 6.67 days) rather than the acute onset often seen in septic arthritis.

The subscapularis muscle sits on the anterior aspect of the scapula behind the chest wall, making surgical approaches to an intramuscular abscess in this location difficult, and often involving extensive dissection of anatomy. The inferomedial approach (*n* = 4) to the subscapularis was used in early reports and involved a longitudinal incision medial and parallel to the medial border of the scapula and dissection of trapezius and rhomboid musculature to gain access to the underlying scapulothoracic space. The more direct deltoid-pectoral approach combined with a conjoint tendon tenotomy (*n* = 3) is more common in recent reports. Christman-Skieller et al. utilised a posterior inferolateral approach to the subscapular space as the bulk of the abscess was near the lateral border of the scapula.^9^ This technique involved an incision along with the superior border of the latissimus dorsi, just inferior to the scapular tip, as well as a small counterincision inferomedial to the scapula through the rhomboid major and serratus anterior muscles to ensure complete drainage of the cavity. This did not involve takedown of the muscle bodies and thus, is the only approach reported that did not also involve invasive dissection of local tendons. Park et al. completed a radical excision of the subscapularis muscle due to suspicions of a suspected sarcoma.^14^ The authors, however, utilised a deltopectoral approach without conjoint tendon tenotomy. The lateral aspect of the subscapularis was split in line with the fibres, minimising any muscle or tendon damage, and the abscess was accessed via blunt finger dissection. This approach not only preserves anatomy but also allows access to wash out the glenohumeral joint and any extension of the infection into the axilla.

This study had several limitations. The retrospective nature of the study and small data set leaves the case series vulnerable to statistical and reporting bias. The data set is also lacking the power to make statistically significant findings, such that any conclusions drawn by the authors is limited and further investigation is required to provide high-quality data for evidence-based guidelines.

From the limited data relating to abscess of the subscapularis muscle, the authors make the following recommendations:

1. Emergency physicians and orthopaedic surgeons should be aware of this unusual presentation of shoulder pain and stiffness.
2. An antra-muscular subscapular abscess may present in paediatric and adult patients.
3. Early diagnosis and treatment is imperative to avoid local spread and haematological dissemination.
4. A subscapular abscess should be treated as a surgical emergency.
5. In unstable patients or when surgical treatment is not immediately available, empirical antibiotics should be started to cover *Staphylococcus aureus*, and in high-risk patients include cover for MRSA.
6. MRI imaging provides a good distinction between muscle tissue and purulent fluid to identify abscess location and extension and plan the surgical approach.
7. In most cases, tendon-sparing approach can be utilised to drain the abscess, including the deltopectoral approach with a subscapularis split, or a direct inferolateral approach.
8. When treated early and appropriately, resolution of symptoms can be expected in the weeks-months after treatment for most patients.

## Conclusion

An intra-muscular subscapularis abscess is an uncommon pathology that can present with vague and subacute shoulder symptoms making diagnosis difficult. They have the potential for local spread and haematogenous dissemination leading to severe clinical disease and deterioration and should be treated as a surgical emergency. *Staphylococcus aureus* is the most commonly cultured organism. Treatment involves surgical drainage and antimicrobial therapy. Historically, surgical drainage has involved aggressive dissection of the local anatomy; however, more recently, tendon-sparing approaches have been described that can access a fluid-filled collection in most locations around the scapula. There are limited data available regarding subscapular abscess, and further investigation is required to provide high-quality evidence to guide treatment decision-making.
